# Successful treatment of inverse psoriasis with ustekinumab: a case report

**DOI:** 10.3389/fmed.2026.1939968

**Published:** 2026-09-09

**Authors:** Yongjun Chen, Jingjing Liu, Yunhan Hu, Ting Yang

**Affiliations:** 1 Department of Dermatology, Huangshi Central Hospital Graduate Joint Training Base, School of Medicine, Wuhan University of Science and Technology, Huangshi, China; 2 The First Clinical School, Hubei Polytechnic University, Huangshi, China; 3 School of Medicine, Wuhan University of Science and Technology, Wuhan, China

**Keywords:** biologics, efficacy, inverse psoriasis, treatment, ustekinumab

## Abstract

Inverse psoriasis is a relatively rare and distinct subtype of psoriasis whose pathogenesis remains incompletely understood. The particular location of lesions presents significant therapeutic challenges, and no consensus guidelines are currently available. Immune mediated inflammation plays a key role in disease development. Lesions are susceptible to riction and moisture, which reduces treatment tolerance in affected areas. We report a case of a 32-year-old male patient with inverse psoriasis successfully treated with ustekinumab. The patient received four subcutaneous injections of ustekinumab, and after 24 weeks of treatment, both skin lesions and pruritus showed marked improvement. Our experience suggests that ustekinumab may represent a novel therapeutic approach for this psoriasis subtype.

## Introduction

Psoriasis is a common chronic inflammatory skin disorder characterized primarily by erythema and scaling. It follows a prolonged course with a tendency to relapse, significantly impairing patients' quality of life (1). Inverse psoriasis, also known as intertriginous psoriasis, represents a distinct subtype that occurs in specific body sites. Lesions affect skin folds including the axillae, inframammary regions, groin, gluteal cleft, genitalia, perineal area, and antecubital fossae. These lesions may be confined to intertriginous areas or coexist with plaques on extensor surfaces (2). Studies report that the prevalence of inverse psoriasis among patients with psoriasis ranges from 3% to 36% (3). Its clinical presentation is often atypical, complicating diagnosis and treatment. Topical therapy remains the mainstay of treatment, with low- to mid-potency corticosteroids generally recommended for short-term disease control and topical calcineurin inhibitors serving as steroid-sparing options for longer-term management (4). However, prolonged corticosteroid exposure in intertriginous areas is limited by the risk of skin atrophy and other local adverse effects, whereas calcineurin inhibitors may cause burning, irritation, and erythema (5). Importantly, some patients experience incomplete or transient responses and frequent relapse after discontinuation of topical therapy, highlighting the need for alternative therapeutic strategies in refractory disease. In the present case, the patient had been treated intermittently with topical corticosteroids and calcineurin inhibitors over the preceding year. Although the lesions and pruritus improved during treatment, they recurred shortly after treatment discontinuation, indicating an inadequate and unsustained response to conventional topical therapy. Ustekinumab, a biologic agent targeting interleukin-12/23, has demonstrated efficacy across various psoriasis forms, including plaque psoriasis (6). It shows particularly favorable outcomes in genital and intertriginous lesions. With its extended half-life, ustekinumab reduces injection frequency and improves treatment adherence, which is crucial for long-term maintenance. Additionally, it is associated with fewer local injection site reactions and lower overall costs.

## Case report

A 32-year-old male presented to our outpatient clinic with a one-year history of “erythema and scaling in both axillae, groin, and scrotum.” One year earlier, he developed erythema, papules, scaling, and pruritus in these areas without any identifiable trigger. He had previously been diagnosed with “eczema” at multiple local hospitals and treated with topical calcineurin inhibitors and corticosteroids. His skin lesions and pruritus improved during treatment but recurred shortly after discontinuation. In the week prior to presentation, both his skin lesions and pruritus worsened, prompting his visit to our clinic. He had been in good health previously, with no history of chronic diseases including hypertension or diabetes, no drug or food allergy history, and no family history of psoriasis.

## Clinical findings

**Figure 1:**
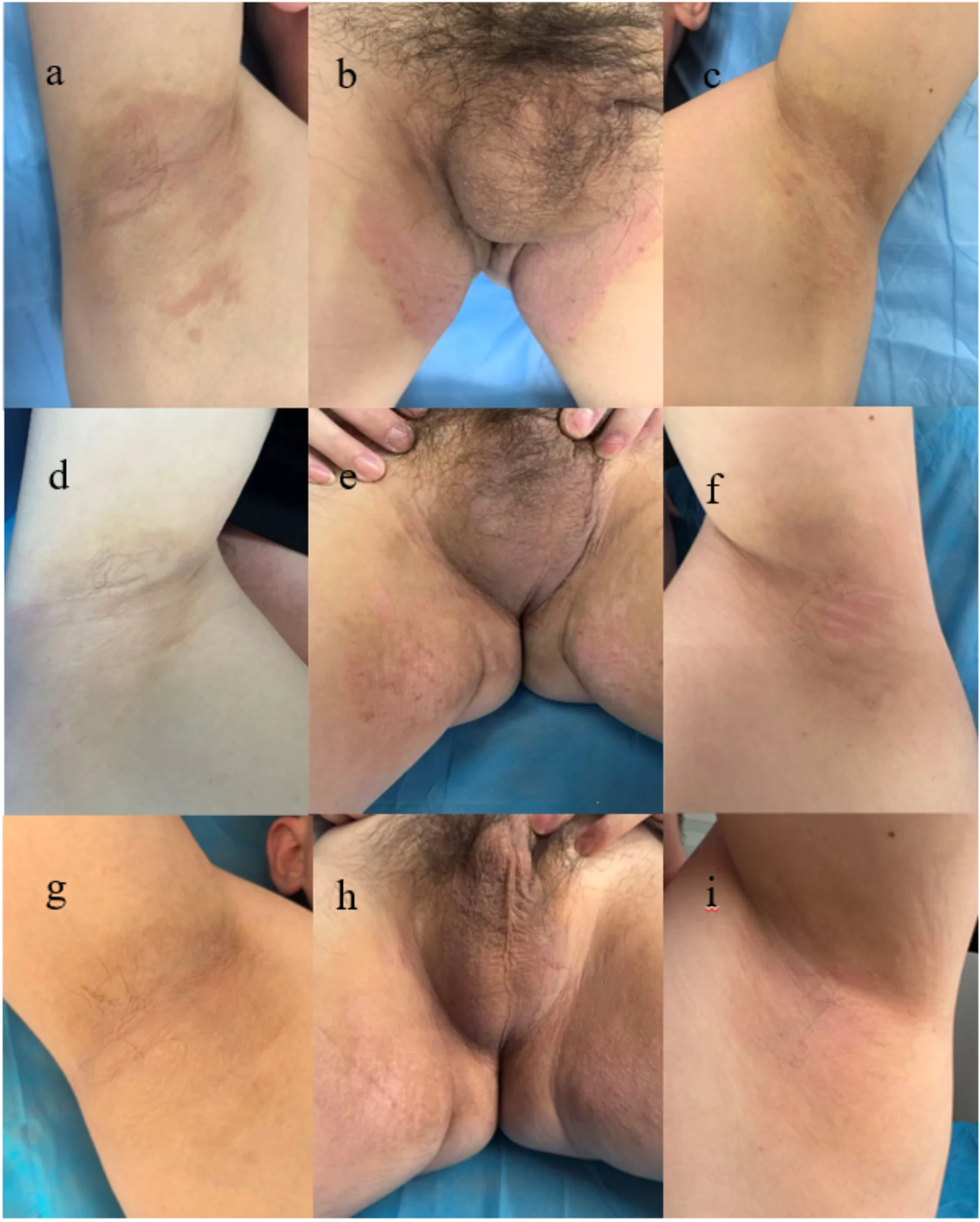
Panels **(a–c)** show the patient before initiation of ustekinumab treatment; panels **(d–f)** at 12 weeks of treatment; and panels **(g–i)** at 24 weeks of treatment.

Dermatologic examination revealed erythema in both axillae, groin, and scrotum, with focal silvery-white scaling and areas of hyperpigmentation and hypopigmentation. Mild induration was palpable on palpation. The scrotum displayed diffuse, well-demarcated erythema accompanied by a positive Auspitz sign and candle-grease sign (Figure 1). The Dermatology Life Quality Index (DLQI) score was 19, the Psoriasis Area and Severity Index (PASI) score was 3, and the Numerical Rating Scale (NRS) for pruritus was 8.

### Diagnostic assessment

**Figure 2:**
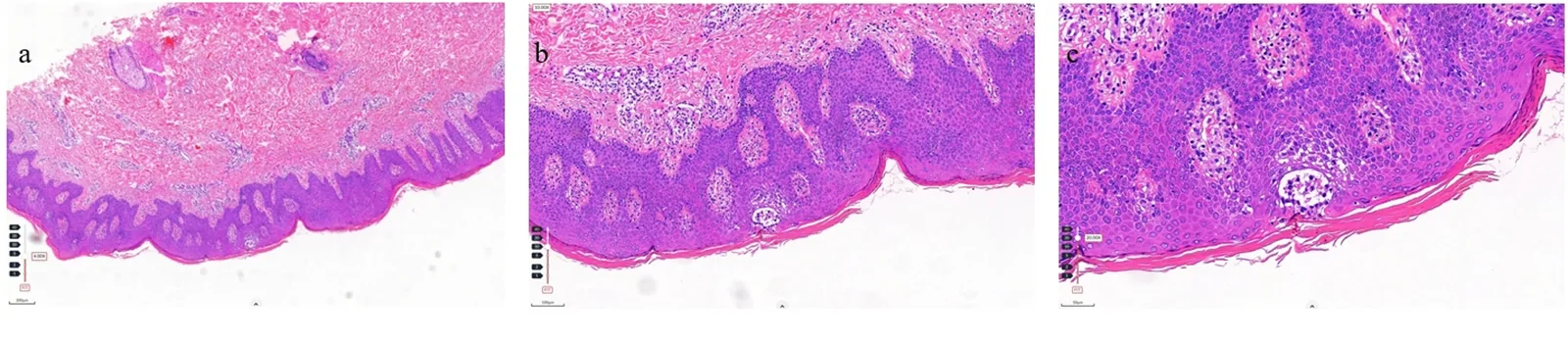
Histopathological examination of skin biopsy specimens. Panels **(a–c)** show hematoxylin and eosin (H&E)-stained sections at  × 4,  × 10, and  × 20 magnification, respectively. Histopathological findings included parakeratosis with Munro microabscess formation, prominent and irregular acanthosis of the stratum spinosum, and prominent perivascular infiltration of chronic inflammatory cells in the superficial dermis.

At our outpatient clinic, the patient underwent laboratory testing and a skin biopsy. Laboratory results, including complete blood count, urinalysis, liver and kidney function tests, coagulation profile, lipid panel, blood glucose, and infectious disease screening, were all within normal limits. Antinuclear antibody screening was negative, as was the fungal *β*-glucan test. Histopathologic examination showed parakeratosis with Munro microabscess formation, acanthosis of the epidermal spinous layer with irregular thickness, and moderate perivascular infiltration of chronic inflammatory cells in the superficial dermis (Figure 2). Because erythematous lesions involving intertriginous areas may resemble infectious diseases, eczema, and other inflammatory dermatoses, several important differential diagnoses were considered in this case (7). Cutaneous candidiasis typically presents with erythema and maceration, often accompanied by satellite papules or pustules. Dermatophyte infections commonly manifest as annular or irregular lesions with an active, scaly advancing border. Seborrheic dermatitis usually presents with relatively ill-defined erythema and greasy scales and frequently involves the scalp, postauricular areas, and other seborrheic sites. Erythrasma, caused by Corynebacterium minutissimum, may present as well-demarcated reddish-brown patches in intertriginous areas and characteristically exhibits coral-red fluorescence under Wood's lamp examination (8). Eczema or contact dermatitis more commonly presents with ill-defined erythema, vesiculation, and exudation, and may be associated with a clear history of exposure to irritants or allergens. In this patient, the bilateral axillae, groins, and scrotum were involved, with focal silvery-white scales, well-demarcated erythema, and positive Auspitz and candle-wax signs, all of which supported a diagnosis of psoriasis. Histopathological examination further demonstrated parakeratosis, Munro microabscesses, acanthosis of the epidermal spinous layer, and perivascular inflammatory cell infiltration in the superficial dermis, providing additional support for the diagnosis of inverse psoriasis. The patient's fungal *β*-D-glucan test was negative. However, a negative *β*-D-glucan result alone cannot completely exclude localized dermatophyte or Candida infection (9). Therefore, this result was not considered sufficient as the sole basis for excluding a fungal infection. When clinical suspicion of fungal infection remains high, direct microscopic examination with potassium hydroxide (KOH) preparation and fungal culture provide more direct diagnostic evidence (10). In the present case, the combination of characteristic clinical features of psoriasis, supportive histopathological findings, and the absence of clinical features strongly suggestive of fungal infection ultimately supported the clinical diagnosis of inverse psoriasis.

## Therapeutic intervention

**Figure 3:**
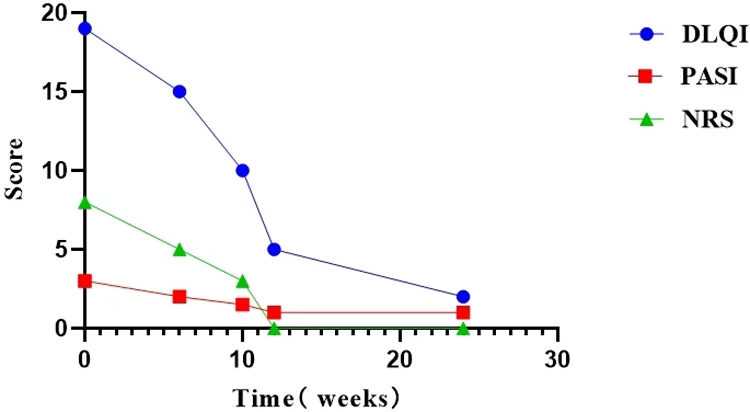
After 24 weeks of treatment, the patient showed marked reductions in the dermatology life quality Index, psoriasis area and severity Index, and numerical rating scale.

Given the patient's inadequate response to conventional topical treatment with corticosteroids and calcineurin inhibitors, together with frequent recurrence after treatment discontinuation, involvement of multiple intertriginous and genital sites, marked pruritus, and substantial impairment in quality of life, escalation to systemic therapy was considered warranted. In addition, given the patient's limited financial resources and the need for an effective and well-tolerated treatment option, ustekinumab was selected in accordance with the latest dichotomous classification of psoriasis, under which systemic therapy is generally recommended for patients with involvement of difficult-to-treat areas (4). Ustekinumab targets the p40 subunit shared by IL-12 and IL-23, thereby preventing their interaction with the IL-12R*β*1 receptor and inhibiting downstream Th1- and Th17-mediated inflammatory pathways. This ultimately suppresses multiple inflammatory processes implicated in the pathogenesis of psoriasis. Ustekinumab has demonstrated favorable efficacy in genital and intertriginous lesions, and its relatively long dosing interval and low incidence of injection-site reactions may be particularly advantageous for patients with disease involving sensitive intertriginous areas (11). Conventional systemic agents such as methotrexate were not considered due to their non-specific anti-inflammatory activity and potential for systemic adverse effects requiring rigorous monitoring of organ function. The patient received subcutaneous ustekinumab at a dose of 45 mg, administered at weeks 0 and 4, followed by maintenance dosing every 12 weeks. By week 24 of treatment, his pruritus had markedly improved, the erythema of the skin lesions had faded, and the lesional area had decreased (Figure 1). His DLQI score improved from 19 to 3, the PASI score decreased from 3 to 1, and the NRS for pruritus dropped from 8 to 0 (Figure 3).

## Follow-up and outcomes

**Figure 4:**
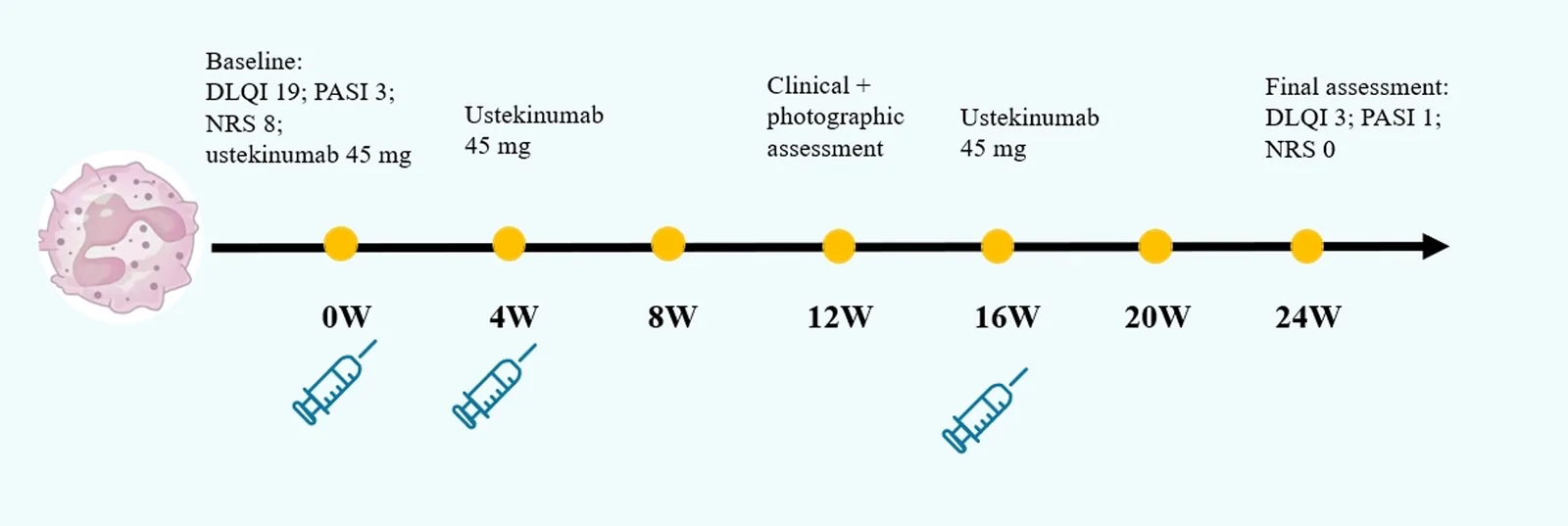
Timeline of ustekinumab treatment initiation.

Figure 4 outlines the timeline from the patient's initial consultation through follow-up. Safety monitoring included monthly assessments of body temperature, complete blood count, and C-reactive protein levels; all results remained within normal ranges, and no infections were reported. The patient expressed satisfaction with the treatment outcome and consented to continue ustekinumab therapy. No adverse events, including infections or allergic reactions, occurred during the treatment period.

## Discussion

Inverse psoriasis, a rare subtype of psoriasis, exhibits distinct features in clinical presentation, pathogenesis, diagnosis, and treatment. Reported prevalence varies widely across studies, ranging from approximately 3% to 36% (7). It predominantly affects intertriginous areas, with the groin being the most commonly involved site. This distribution is closely tied to the unique microenvironment of skin folds, characterized by warmth, humidity, hyperhidrosis, and friction, which can compromise the skin barrier and provoke inflammation. Moreover, flexural skin inherently has a weaker barrier and heightened sensitivity, which may amplify the underlying immune-inflammatory cascade and abnormal keratinocyte proliferation characteristic of psoriasis, ultimately favoring lesion localization in these regions (12). Compared with plaque psoriasis, inverse psoriasis is associated with greater impairment in health-related quality of life, particularly due to pruritus and feelings of shame. Diagnosis relies primarily on clinical findings, supported by skin biopsy and fungal testing to exclude secondary infection or mimickers. In cases with atypical or isolated symptoms, biopsy can provide critical diagnostic clarification. In this patient, erythema involving the axillae, groin, and scrotum, with focal silvery-white scaling, provided key initial diagnostic clues.

The pathogenesis of inverse psoriasis overlaps substantially with that of plaque psoriasis, centered on dysregulated activation of the IL-23/IL-17 axis. IL-23 drives Th17 cell differentiation, leading to secretion of pro-inflammatory cytokines such as IL-17, which stimulate keratinocyte proliferation and inflammation, resulting in erythema and epidermal hyperplasia. Common histopathologic features include eosinophilic infiltration, epidermal spongiosis, and focal serous exudation beneath scales (2). Our patient's biopsy revealed classic changes consistent with inverse psoriasis, reinforcing the diagnostic utility of histopathology in this condition.

Treatment generally follows a stepwise approach. First-line therapy consists of topical agents, typically low- to mid-potency corticosteroids and calcineurin inhibitors (e.g., tacrolimus). Adjunctive options may include topical retinoids, salicylic acid, excimer laser therapy, and botulinum toxin injections (7). Importantly, because affected areas have thinner, more sensitive skin often under occlusion, therapeutic strategies differ markedly from those used for chronic plaque psoriasis; lower-potency steroids or calcineurin inhibitors are preferred to minimize local adverse effects (13). Although several IL-17 and IL-23 inhibitors have emerged as promising therapeutic options for inverse psoriasis in recent years (14, 15), ustekinumab was selected in the present case based on a comprehensive assessment of treatment efficacy, convenience, the patient's occupational circumstances, long-term treatment adherence, and affordability. The patient had previously received topical corticosteroids and calcineurin inhibitors, but the response was inadequate and difficult to sustain, with recurrent lesions involving multiple intertriginous sites and significant pruritus. According to the latest two-tiered treatment approach for psoriasis, early initiation of systemic therapy should be considered in such patients. Ustekinumab exerts its therapeutic effects by targeting the shared p40 subunit of IL-12 and IL-23. By binding to the p40 subunit, ustekinumab blocks IL-12-mediated Th1-cell activation and suppresses the downstream production of proinflammatory cytokines, including TNF-α and IL-2. It also inhibits IL-23-driven Th17-cell activation and reduces IL-17A production (16). These cytokines act on various cell types, including keratinocytes and epithelial cells. By suppressing these inflammatory pathways, ustekinumab can alleviate cutaneous inflammation and thereby improve erythema, scaling, and other psoriasis-related clinical manifestations. For this patient, ustekinumab was selected based on a patient-centered, comprehensive assessment rather than solely on the presumed efficacy of a particular agent. The patient had inadequate disease control with topical corticosteroids and calcineurin inhibitors and experienced frequent recurrence after treatment discontinuation. In addition, multiple intertriginous sites were involved and accompanied by significant pruritus, suggesting that topical therapy alone was insufficient to achieve sustained disease control. Therefore, escalation to biologic therapy was considered reasonable after careful consideration of disease severity, previous treatment response, and the patient's therapeutic needs. The choice of ustekinumab was based on a comprehensive assessment of expected efficacy, dosing convenience, the patient's occupation and long-term work away from home, treatment adherence, and financial burden. Ustekinumab has well-established efficacy and safety data in psoriasis and has also been used in the management of special-site psoriasis. Moreover, its relatively infrequent dosing schedule, with administration at weeks 0 and 4 followed by subcutaneous administration every 12 weeks, reduces the need for frequent medical visits and treatment-related time costs. This dosing regimen may be particularly advantageous for patients who work away from home and may facilitate long-term treatment adherence. From a pharmacoeconomic perspective, ustekinumab is currently the least expensive biologic therapy for psoriasis in China. In addition to direct medication costs, factors such as dosing frequency, healthcare resource utilization, travel and time costs associated with medical visits, and long-term treatment adherence should also be considered. In this case, the relatively infrequent dosing schedule and local accessibility of ustekinumab may help reduce the indirect costs and time burden associated with frequent medical visits during long-term treatment. Therefore, after balancing efficacy, safety, treatment convenience, and long-term economic considerations, ustekinumab was considered an appropriate individualized treatment option for this patient. Therefore, the selection of biologic therapy should be individualized according to disease characteristics, previous treatment response, patient preferences, occupational circumstances, treatment accessibility, dosing convenience, and economic considerations. However, it should be emphasized that large randomized controlled trials specifically evaluating biologic therapies for inverse psoriasis remain lacking. Future head-to-head comparative studies of different biologic agents are warranted to determine the optimal treatment strategy for this challenging clinical phenotype.

## Conclusion

Inverse psoriasis exhibits distinct clinical features, and its management remains challenging due to the absence of standardized, evidence-based therapeutic guidelines. In this case, 24 weeks of ustekinumab therapy led to marked improvement in lesions and pruritus affecting the axillae, groin, and scrotum, along with a substantial reduction in the DLQI score, underscoring its therapeutic potential. These findings offer clinicians a valuable reference for treating similar patients who have responded inadequately to conventional therapies. Given the scarcity of relevant studies, multicenter, large-sample, long-term follow-up trials are warranted to further evaluate the durability, efficacy, and safety of ustekinumab and to refine treatment strategies for inverse psoriasis.

## Data Availability

The original contributions presented in the study are included in the article/Supplementary Material, further inquiries can be directed to the corresponding author.
